# Ilaprazole-amoxicillin dual therapy at high dose as a first-line treatment for helicobacter pylori infection in Hainan: a single-center, open-label, noninferiority, randomized controlled trial

**DOI:** 10.1186/s12876-023-02890-5

**Published:** 2023-07-24

**Authors:** Xiao-Dong Zhang, Da-Ya Zhang, Run-Xiang Chen, Shi-Ju Chen, Chen Chen, Fan Zeng, Shi-Mei Huang, Da Li, Fei-Hu Bai

**Affiliations:** 1 Graduate School, Hainan Medical University, Haikou, China; 2 Department of Gastroenterology, The Second Affiliated Hospital of Hainan Medical University, Yehai Avenue, #368, Longhua District, Haikou, Hainan Province China; 3 The Gastroenterology Clinical Medical Center of Hainan Province, Haikou, China

**Keywords:** *Helicobacter pylori*, Dual therapy, Quadruple therapy, Ilaprazole, Eradication rate, Noninferiority

## Abstract

**Objectives:**

This study aimed to evaluate the efficacy, adverse events, patient compliance, and cost of dual therapy with Ilaprazole-amoxicillin (IA) at high dose versus Ilaprazole-amoxicillin-furazolidone-bismuth (IAFB) quadruple therapy for the Helicobacter pylori (*H.pylori*) infection among Chinese patients.

**Methods:**

200 patients who had tested positive for *H. pylori* and undergoing upper gastrointestinal endoscopy after being diagnosed with chronic gastritis participated in this open-label randomized controlled clinical trial. Patients were randomized to Group A and Group B: the 14-day IA dual treatment group (101) and IAFB quadruple treatment group (99). The ^13^ C urea breath test was conducted to determine whether *H. pylori* had been eliminated 4–6 weeks after the treatment. Eradication rates, drug-related adverse events, patient compliance, and drug costs were compared between the two treatment groups.

**Results:**

Eradication rates in group A were 92.1% and 94.9%, depending on the intention-to-treat (ITT), per-protocol (PP), respectively, which was similar to group B (91.9% and 93.6%). There was no significant difference observed in adverse events between the two groups (*P* = 0.518). Interestingly, compliance was significantly higher in group A compared to the group B (*P* = 0.031). In addition, drug costs were significantly lower for group A in comparison to the group B.

**Conclusions:**

IA dual therapy was found to be equally effective, safer and less costly than IAFB quadruple therapy. Therefore, these therapies can be potentially considered as first-line regimens for empirical treatment.

## Introduction

Helicobacter pylori (*H. pylori*) has been identified as a major cause of the development of chronic gastritis, peptic ulcer, gastric Mucosa-associated lymphoid tissue (MALT) lymphoma and gastric cancer [1]. Hence, in the expert consensus such as the Maastricht VI Consensus, the Toronto Consensus and the 6th National Consensus Report on the Management of *H. pylori* Infection, 14-day bismuth-containing quadruple therapy was recommended as the treatment of choice for management of infection [1]. The consensus clearly stated that *H. pylori* eradication can effectively promote peptic ulcer healing and reduce the incidence of gastric cancer as well as ulcers, and that *H. pylori* eradication can lead to remission in 80% of patients with early gastric MALT lymphoma [1]. *H. pylori* eradication has been found to be effective in reducing socioeconomic stress [1–3]. Long-term use of multiple antibiotics can significantly enhance the resistance of *H. pylori* [4]. For instance, the resistance rate of metronidazole in China has been reported to be as high as 40-70%, whereas the resistance rate of both clarithromycin and levofloxacin is 20-50% [4]. The eradication rate of bismuth-containing quadruple therapy has been reported to decrease substantially concomitant with the increase of antibiotic resistance, but the incidence of adverse events (AEs) of quadruple therapy remains relatively high [4]. Therefore, there is an urgent need for development of novel treatment option that can reduce AEs and antibiotic use. A number of studies have shown that diphtherapy with high-dose amoxicillin combined with PPI can be potentially used as a first-line or remedial therapy for *H. pylori* eradication, with eradication rates of more than 90%, even higher than those of bismuth-containing quadruple therapy [5–7]. Ilaprazole is a proton pump inhibitor belonging to benzimidazole class that can irreversibly inhibit enzymes on H+ -K+ -ATP and cause inhibition of gastric acid production [8]. It has been established that compared to the first generation PPI, it has the advantages of long half-life, faster and stronger acid inhibition and is not affected by CYP2C19 gene polymorphism during metabolism [9, 10]. Moreover, Ilaprazole enteric tablets are widely used, which can be more easily absorbed and thus exert a stronger acid-suppressive effect [11]. In addition, prior reports have shown that the rate of AEs of Ilaprazole was relatively low in H pylori-infected patients aged between 14 and 70 years old thus indicating its relative safety and reliability [12, 13]. However, the efficacy of dual therapy with Ilaprazole (Active Ingredient: Eprazole; Lizhu Group Lizhu Pharmaceutical Factory; Zhujiang City, Guangdong Province, China)-amoxicillin (IA) at high dose has not been reported so far. Hence, we sought to determine whether dual therapy with IA in this study could improve the clinical outcomes, avoid unnecessary AEs, and also reduce the cost as a first-line treatment option for *H. pylori* eradication in Chinese patients.

## Patients and methods

### Patients and study design

Initial screening of patients aged ≥ 14 years who were diagnosed positive for *H. pylori* by ^13^ C urea breath test (UBT) was conducted at the Second Affiliated Hospital of Hainan Medical University in the period ranging from January 2022 to December 2022 in a specialized outpatient clinic for *H. pylori* diagnosis. The study ultimately included 200 patients who had tested positive for *H. pylori* while undergoing upper GI endoscopy as a result of chronic gastritis. The patients were randomly divided into Group A and Group B: the 14-day Ilaprazole-amoxicillin (IA) dual treatment group (101) and Ilaprazole-amoxicillin-furazolidone-bismuth (IAFB) treatment group (99). The ^13^ C UBT indicated that *H. pylori* had been eliminated 4–6 weeks after treatment. *H. pylori* eradication rates were determined by intention-to-treat (ITT) and per-protocol (PP) analysis. In addition, eradication rates, drug-related AEs, patient compliance, and drug costs were compared between the two groups.

If the subject failed the initial treatment, the patient was recommended for the remedial treatment after 6 months of outpatient follow-up. Patients in group A who failed *H. pylori* eradication were treated with remedial treatment using IAFB treatment administered in group B, whereas those in group B were treated using IA regimen from group A. For patients who failed the second treatment, three gastric mucosal samples, including one sinus sample and two gastric body samples, were collected after the gastroscopy. Three samples were collected and placed in *H. pylori* isolation and preservation tubes containing the brain heart infusions. The *H. pylori* positive strains were then tested by bacterial culture and drug sensitivity test for clarithromycin, amoxicillin, levofloxacin, furazolidone, tetracycline and metronidazole. The drug sensitivity test was performed by the paper diffusion method. The susceptibility, resistance or mediating effect of each sample was also assessed according to the size of the zone of inhibition (different antibiotics might have different criteria for the size of the zone of inhibition).

The detailed exclusion criteria used were as follows: (1) pregnant or lactating women; (2) with severe concomitant disease, malignancy; (3) history of allergy to the study drug; (4) PPI or antibiotics administered within the previous month; (5) bleeding from a severe ulcer; (6) patients previously treated with anti-*H. pylori* drug. The subjects were instructed by investigators to complete a questionnaire about *H. pylori*, which included the gender, age, race, education level, area of residence, lifestyle, dietary habits, relevant information about their family members and their past medical history.

This clinical trial study was reviewed and approved by the Ethics Committee of the Second Affiliated Hospital of Hainan Medical University (No. LW2021038). A written informed consent was obtained from all the participants and also from legal guardians of those participants whose age was below 16 years of age. In addition, this study was conducted in accordance with the Declaration of Helsinki and other relevant regulations. This single-center, open-label, noninferiority, randomized controlled trial has been registered with the China Clinical Trials Registry (www.chictr.org.cn) to evaluate the effect of dual therapy with IA at high dose versus IAFB quadruple therapy as first-line treatment for *H. pylori* infection in Chinese patients. The first trial registration was done on 24/10/2021 (ChiCTR2100052308).

### Calculation of sample size

**Fig. 1:**
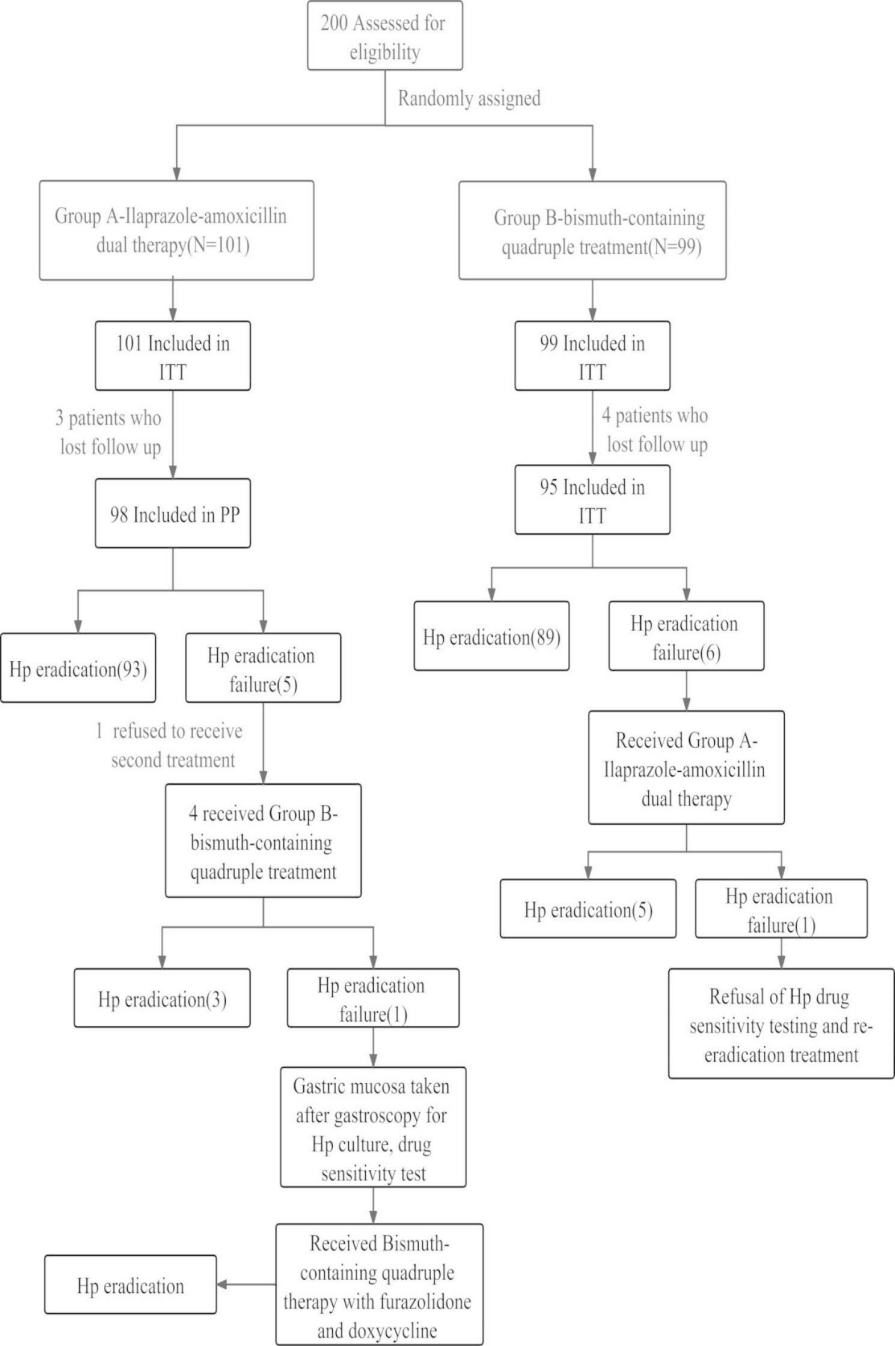
A Schematic diagram representing the study design and results

Based on the previous literature, the eradication rate of *H. pylori* was 91.2% [14]. In this study, a randomized controlled trial with a non-inferiority design was conducted, assuming an expected *H. pylori* eradication rate of 91.2% in both the groups, a non-inferiority threshold of 5%, α = 0.025 (unilateral), certainty (1-β) = 0.9, and ratio of the number of cases in the test group to the number of controls = 1:1. The sample sizes of the test and control groups were calculated using “Non- Inferiority Tests for the Difference Between Two Proportions” in the PASS 2021 software. Overall, 89 *H. pylori*-infected patients were required in the test and control groups respectively, considering a 10% failure rate, and finally 200 patients with *H. pylori*-positive chronic gastritis were needed. Thereafter, 200 subjects were simply randomly assigned into two different treatment groups (Fig. 1). All the enrolled patients were included in the ITT analysis, and those with unconfirmed *H. pylori* eradication status due to follow-up failure were categorized as the treatment failures in the ITT analysis. However, the patients for whom *H. pylori* eradication status data were not available were excluded from the PP analysis due to lack of follow-up data.

### Drug information

Ilaprazole Enteric Tablets (Active Ingredient: Eprazole; Lizhu Group Lizhu Pharmaceutical Factory; Zhujiang City, Guangdong Province, China), Bismuth Citrate Potassium Capsules (Active Ingredient: Bismuth Citrate Potassium; Hunan Huana Pharmaceutical Factory Company Limited; Changsha City, Hunan Province, China), Amoxicillin Capsules (Active Ingredient: Amoxicillin; Hainan General Sanyo Pharmaceutical Co); Furazolidone Tablets (Active ingredient: Furazolidone; Shanxi Yunpeng Pharmaceutical Co., Ltd; Xianfen, Shanxi Province, China) were used in this study.

### Treatments and follow-up

The IA-14-day group consisted of Ilaprazole (5.0 mg twice daily, half an hour before meals) and amoxicillin capsules (1 g three times daily, half an hour after meals) for 14 days. The IAFB-14-day group comprised of Ilaprazole (5.0 mg twice a day half an hour before meals), bismuth citrate potassium capsules (120.0 mg twice a day half an hour before meals), amoxicillin capsules (1 g twice a day half an hour after meals), and furazolidone tablets (0.1 g twice a day half an hour after meals) for 14 days. The patients were given medication instructions during the course of the treatment and were followed up by phone on day nine or ten to determine their medication experience. The patients were instructed not to smoke, drink alcohol or eat acidic food during the treatment and to stop intake of the drug for one month after the 14-day course, with outpatient follow-up to investigate patient compliance and AEs.

### Outcome measurements

The primary outcome of this study was to determine the potential efficacy of IA dual therapy in comparison to IAFB quadruple therapy for *H. pylori* eradication rates using the 13 C-UBT assay. A threshold value greater than 2.4 defined patients with *H. pylori* and indicated failure of eradication therapy. The secondary outcomes consisted of medication compliance, AEs and cost associated with different drugs. The Compliance was evaluated 1–3 days after the treatment. Medication possession rate (MPR) was defined as the proportion of days during a fixed 14-day treatment that the patients had access to medication, which was measured by patient pill counts. 80% of MPR was considered as a good compliance. AEs, was classified as “mild” (discomfort without interruption of the daily activities), “moderate” (interruption of their daily activities to some extent) and “severe " (severe interruption of the daily activities). The cost of drugs for both the treatment options were calculated according to the 2022 Hainan Drug Pricing Catalogue. The prices were expressed in U.S. dollars, with Chinese currency converted to U.S. dollars at the exchange rate published in January 2022 was: $1.00 = RMB 6.3588.

### Statistical analysis

The various baseline characteristics, *H. pylori* eradication rates, AEs, and treatment compliance of patients were analyzed using chi-square tests, Fisher’s exact test and ANOVA. Noninferiority was established if 95% lower confidence limit for the difference in eradication rates between IA dual therapy and IAFB quadruple therapy was > -0.14 with one-sided alpha level of 0.025. In addition, the different factors associated with *H. pylori* eradication were also analyzed. p values less than 0.05 were considered as statistically significant.

## Results

### Baseline demographic and clinical characteristics of the study subjects

There were no significant differences found in the sociodemographic data between the two groups (*P* > 0.05) (Table 1).

**Table 1:** Baseline demographics and clinical characteristics of the study subjects between Group A and Group B

	Group A (n = 101)	Group B (n = 99)	χ2/t	*P*
Gender			0.504	0.484
Male	52(51.5%)	46(46.5%)		
Female	49(48.5%)	53(53.5%)		
Age (yr)	41.14 ± 12.784	43.99 ± 14.718	1.158	0.247
Ethnicity			-	0.683
Han	97(96.0%)	97(98.0%)		
Other ethnicity	4(4.0%)	2(2.0%)		
Place of residence			2.969	0.090
Downtown	50(49.5%)	61(61.6%)		
County or Township	51(50.5%)	38(38.4%)		
Personal monthly income			0.060	0.868
< 5000	78(77.2%)	75(75.8%)		
≥ 5000	23(22.8%)	24(24.2%)		
Education status			0.660	0.451
High school or less	66(65.3%)	70(70.7%)		
College or more	35(34.7%)	29(29.3%)		
Drinking water			5.536	0.065
Tap water	76(75.2%)	85(85.9%)		
Purified water	15(14.9%)	5(5.1%)		
Well water	10(9.9%)	9(9.1%)		
Brushing times			-	1.000
1 ~ 2	97(96.0%)	95(96.0%)		
≥ 3	4(4.0%)	4(4.0%)		

Data are expressed as mean ± sd; categorical data are presented as number of subjects and percentage in parentheses

### Comparison of eradication rates between Group A and Group B

It was found that during ITT analysis, eradication rate was 92.1% (93/101; 95% CI 93.4%-98.7%) in Group A and 91.9% (91/99; 95% CI 93.2%-98.87%) in the Group B (Table 2). In the PP analysis, eradication rate was 94.9% (93/98; 95% CI 95.2%-99.7%) in the Group A and 93.6% (89/95; 95% CI 96.2%-97.4%) in the Group B. There was no significant difference in eradication rates between the two groups (*P* = 1.000 and *P* = 0.765 in ITT and PP analyses, respectively).

**Table 2:** Eradication rates of H. pylori infection between Group A and Group B

	Group A	Group B	χ2	*P*
ITT	92.1% (93/101)	91.9% (91/99)	0.002	1.000
95% CI	93.4-98.7%	93.2-98.7%		
PP	94.9% (93/98)	93.6% (89/95)	0.132	0.765
95% CI	95.2-99.7%	96.2-97.4%		

CI, confidence interval; ITT, intention-to-treat; PP, per-protocol

### Drug-induced AEs, patient compliance, and drug cost between Group A and Group B

The AEs that occurred during eradication therapy included nausea, diarrhea, dizziness, taste changes, rash, and black stools, etc. As depicted in Table 3, there was no significant differences in treatment-related AEs between group A and group B (13.9% and 17.2%, respectively). The compliance was significantly higher in the group A (95.1%, 96/101) than in the group B (87.9%, 85/99) (*P* = 0.031). The cost for group A was $51.67 ($50.32 for 28 Ilaprazole enteric tablets and $1.35 for 168 amoxicillin capsules), which was relatively less costly compared with $55.71 for group B ($50.32 for 28 Ilaprazole enteric tablets, $3.21 for 28 sachets of bismuth potassium citrate capsules, $0.90 for 112 amoxicillin capsules, and $1.28 for 280 furazolidone).

**Table 3:** Drug-induced adverse effects and patient compliance between Group A and Group B

	Group A (n = 101)	Group B (n = 99)	χ2	*P*
Adverse events	14/101(13.9%)	17/99(17.2%)	0.418	0.562
Abdominal distension	0(0%)	3(3.0%)	-	0.119
Diarrhea	2(2.0%)	0(0%)	-	0.498
Nausea	4(4.0%)	3(3.0%)	-	1.000
Dizziness	4(4.0%)	3(3.0%)	-	1.000
Palpitations	1(1.0%)	2(2.0%)	-	0.619
Skin rash	1(1.0%)	2(2.0%)	-	0.619
Black stool	1(1.0%)	2(2.0%)	-	0.619
Constipation	1(1.0%)	2(2.0%)	-	0.619
Compliance	96/101(95.1%)	85/99(87.9%)	4.912	0.031

Compliance was indicative of patients who took at least 80% of study drugs

### Factors associated with ***H. pylori*** eradication

The eradication rate of Group A was associated with often washing hands after urinating (*P* = 0.042). However, various other important factors such as gender, utensils for public use, consumption of fruits and vegetables, family history of *H. pylori* infection and gastrointestinal (GI) symptoms etc. were not observed to be associated with the eradication rate of *H. pylori* in the univariate correlation analysis in Group A or Group B or Group A + B (*P* > 0.05) (Table 4). When multifactorial analysis was conducted, we used gender in for correction and did not find a single meaningful variable in Group A (Table 5).

**Table 4:** Factors affecting eradication of *H. pylori* infection

	Group A (n = 98)	Group B (n = 95)	Group A + B (n = 193)
Gender	*P* = 0.367	*P* = 0.211	*χ2 = 0.086,P = 1.000*
Male	48/52(92.3%)	43/44(97.7%)	91/96(94.8%)
Female	45/46(97.8%)	46/51(90.2%)	91/97(93.8%)
ethnicity	*P* = 0.192	*P* = 1.000	*P* = 0.3
Han	90/94(95.7%)	87/93(93.5%)	177/187(94.7%)
Other ethnicity	3/4(75%)	2/2(100%)	5/6(83.3%)
Place of residence	*P* = 0.364	*P* = 0.403	*χ2 = 0.000, P = 1.000*
Downtown	46/47(97.9%)	54/59(91.5%)	100/106(94.3%)
County or Township	47/51(92.2%)	35/36(97.2%)	82/87(94.3%)
Education status	*P* = 0.345	*P* = 1.000	*χ2 = 0.316, P = 0.574*
High school or less	61/63(96.8%)	62/66(93.9%)	123/129(9.3%)
College or more	32/35(91.4%)	27/29(93.1%)	59/64(92.2%)
Personal monthly income	*P* = 1.000	*P* = 0.135	*χ2 = 0.539 P = 0.463*
< 5000	72/76(94.7%)	70/73(95.9%)	142/149(95.3%)
≥ 5000	21/22(95.5%)	19/22(86.4%)	40/44(90.9%)
Utensils for public use	*P* = 1.000	*P* = 0.670	χ2 = 0.000, *P* = 1.000
Often	62/66(93.9%)	56/59(94.9%)	118/125(94.4%)
Rarely	31/32(96.9%)	33/36(91.7%)	64/68(94.1%)
Washing hands after urinating	*P* = 0.042	*P* = 0.344	χ2 = 0.095, *P* = 0.758
Often	78/80(97.5%)	70/76(92.1%)	148/156(94.9%)
Rarely	15/18(83.3%)	19/19(100%)	34/37(91.9%)
Consumption of fruits and vegetables	*P* = 1.000	*P* = 0.408	*χ2 = 0.063 P = 0.802*
Often	45/47(95.7%)	55/60(91.7%)	100/107(93.5%)
Rarely	48/51(94.1%)	34/35(97.1%)	82/86(95.3%)
History of H. pylori infection in family members	*P* = 0.321	*P* = 1.000	χ2 = 0.611 *P* = 0.434
Yes	26/26(100%)	18/19(94.7%)	44/45(97.8%)
No	67/72(93.1%)	71/76(93.4%)	138/148(93.2%)
Gastrointestinal symptoms	*P* = 0.334	*P* = 1.000	*χ2 = 0.165 P = 0.685*
Yes	72/75(96.0%)	47/50(94.0%)	119/125(95.2%)
No	21/23(91.3%)	42/45(93.3%)	63/68(92.6%)
Eating betel nut	*P* = 0.129	*P* = 1.000	χ2 = 0.033 *P* = 0.856
Yes	11/13(84.6%)	10/10(100%)	21/23(91.3%)
No	82/85(96.5%)	79/85(92.9%)	161/170(94.7%)
Drinking water	*P* = 1.000	*χ2 = 0.302, P = 1.000*	*χ2 = 0.740, P = 0.844*
Tap water	70/74(94.6%)	75/81(92.6%)	145/155(93.5%)
Purified water	13/14(92.9%)	5/5(100%)	18/19(94.7%)
Well water	10/10(100%)	9/9(100%)	19/19(100%)
Brushing times	*P* = 1.000	*P* = 1.000	*P* = 1.000
1 ~ 2	89/94(94.7%)	85/91(93.4%)	174/185(94.1%)
≥ 3	4/4(100%)	4/4(100%)	8/8(100%)
History of gastric cancer in immediate family	*P* = 1.000	*P* = 1.000	*P* = 1.000
Yes	7/7(100%)	2/2(100%)	9/9(100%)
No	86/91(94.5%)	87/93(93.5%)	173/184(94.0%)

**Table 5:** Multifactorial analysis of factors associated with H. pylori infection successful eradication in Group A

Factors	β	S.E.B	Wald χ2	P	OR (95%CI)
Female	20.976	4578.514	0	0.996	1,287,017,905
Often washing hands after urinating	-21.336	4578.514	0	0.996	0

### Remedial therapy in patients with ***H. pylori*** eradication failure

It was observed that there were five patients in group A who failed *H. pylori* eradication, one of whom refused a second treatment and four of whom received IAFB treatment from the group B. Overall, In the end, 3 cases were successfully eradicated, but in 1 case eradication was not successful. This patient with second failed eradication was found to be resistant to clarithromycin, levofloxacin, and metronidazole, but sensitive to amoxicillin, furazolidone, as well as doxycycline receiving bismuth-containing quadruple therapy with furazolidone and doxycycline and the *H. pylori* infection was finally eradicated successfully.

However, in group B, 6 patients who failed *H. pylori* eradication received IA dual therapy from group A. Ultimately, 5 case were successfully eradicated but 1 case failed to eradication. This patients with second failed eradication refused to *H. pylori* drug sensitivity testing and re-eradication treatment.

## Discussion

With bismuth-containing quadruple therapy emerging as the first-line therapy for the eradication of *H. pylori*, the rate of *H. pylori* eradication has decreased primarily because multiple drug compositions might increase the incidence of AEs, thereby decreasing the patient compliance and leading to failure of *H. pylori* eradication [15]. It has been established that especially in older age groups, poor tolerance of AEs as well as the more complex and less easily understood drug instructions for all the four drugs can result in poor patient compliance and lead to *H. pylori* eradication failure [16, 17].

In addition, compliance with medication is also critical for the successful eradication of *H. pylori*. We further recognize that dosing times is also important for compliance. Tid regimen (Group A) was administered three times a day, which could have effectively increased patient burden and reduced compliance. However, we still believe that the tid regimen (Group A) might be relatively better than the bid regimen (Group B). Our diphtherapy was administered three times a day, coinciding with the number of daily meals, which may increase patient compliance, but of course did not help patients with irregular diets. In addition, there are several important factors that can potentially affect patient compliance, such as the complexity of the medication administered as well as the side effects of the medication. Multiple drug compositions in bid regimen (Group B) might also increase the incidence of adverse reactions and thereby reduce patient compliance. It has been found that especially in the elderly population, poor tolerance of adverse reactions and the more complex and less easily understood drug descriptions of the four drugs might contribute to the poor patient compliance and can in turn lead to failure of *H. pylori* eradication. Tid regimen (Group A) only consists of two main drugs, high-dose amoxicillin, which acts primarily as a bactericidal agent, and PPI, which functions to maintain a high pH in the stomach. Amoxicillin can exhibit a robust and stable bactericidal effect when its blood concentration is approximately ten times higher than the minimum inhibitory concentration (MIC) [18, 19]. Hence, in order to achieve a high eradication rate of *H.pylori* and to maintain a sufficiently high blood concentration, we decided to administer a single dose of 1 g amoxicillin with a dosing frequency of three times a day. For PPI, we selected Ilaprazole, which has a relatively longer acid inhibition time and is not affected by *CYP2C19* gene polymorphism [9, 10]. There are few prior reports related to 7-day or 10-day *H. pylori* regimens, especially regimens containing Ilaprazole diphtheria or quadruple therapy. The effect of the 10-day regimen and the 7-day regimen can be investigated in detail in the future. In recent years, vonorasen fumarate has gained significant attention as a new acid inhibitor and its inhibitory effect on gastric acid has been found to be stronger than that of PPI [20]. Vonorasen diphtherapy has the advantages of high eradication rate of *H. pylori*, low rate of AEs, short course of treatment, less combination of drugs, reduced impact on intestinal microbiota in comparison with the traditional quadruple therapy containing PPI [21]. At present, there are few studies describing the impact of vonorazole in China, especially the effect of vonorazole on high-dose diphtherapy as well as quadruple therapy still needs to analyzed in depth.

We also observed in our study, that regularly washing hands after urinating was correlated with the success of *H. pylori* eradication in IA dual therapy. When we conducted a multifactorial analysis, we selected gender [16] in for correction and did not find a single meaningful variable, which could be directly related to the small total sample size and the large difference in sample size between the eradication success and failure groups. In addition, we also combined the analysis of IA dual therapy and quadruple therapy into two different groups, with 182 cases in the eradication success group and 11 cases in the failure group, to identify meaningful factors for *H.pylori* eradication success, but ultimately no meaningful factors were found. Hence, future multicenter clinical studies could be conducted to further clarify the different factors involved.

It may be possible that betel nut chewing can alter the oral and gastric PH, affect the colonization of normal flora and decreases saliva secretion, thereby causing the local bactericidal effect of the oral cavity weaker and significantly increasing the risk of *H. pylori* infection [22]. In our study, betel nut chewing was not found to be correlated with the failure of *H. pylori* eradication in both groups. The relevant mechanism underlying this observation still needs to be further studied.

The efficacy of our bismuth-containing quadruple therapy was relatively high and similar to those reported in some previously performed studies in China [23, 24]. The success of *H.pylori* eradication could be influenced by several factors, including acid inhibition level, antibiotic resistance, patient compliance, host factors and bacterial factors [15, 25]. Moreover, various host related factors such as gender, old age, smoking, ethnicity, *H.pylori* colonization site, and diabetes have been associated with *H.pylori* eradication failure [15, 25]. We have carried out *H.pylori* culture and drug sensitivity in the past in Hainan and found that *H.pylori* was more sensitive to amoxicillin, furazolidone, and tetracycline and resistant to metronidazole. In this study, amoxicillin, furazolidone, Ilaprazole, and bismuth potassium citrate were selected for the bismuth-containing quadruple therapy based on excellent superiority [9, 10, 20]. A number of prior studies have demonstrated that bismuth used in the treatment of H. pylori has a short cycle time, low absorption rate, and relatively high clinical safety [12, 26–28]. A meta-analysis conducted by Alexander C Ford et al. reported that bismuth can increase the susceptibility of *H. pylori* to antibiotics and has a high safety and tolerability profile for *H pylori*-infected patients over the age of 16 years [28]. Here are other reasons for our high quadruple eradication rate. The course of treatment can also affect the rate of H. pylori eradication. For instance, in PPI-based quadruple therapy, the effect of 14 days was significantly higher than that of 7 and 10 days [29, 30]. Our treatment reached enough time for H. pylori eradication. Diabetes has also been associated with H. pylori eradication failure. Our study population excluded people with chronic diseases such as those affected with concomitant diabetes and hypertension. Smoking serves as an unfavorable factor for the failure of H. pylori eradication because it not only stimulates the secretion of gastric acid, but can also substantially reduce the secretion of bicarbonate from the duodenum and pancreas, thereby resulting in a pH of < 6 in the stomach, which can negatively affect the therapeutic effect [31]. Before initiation of the treatment regimen, we instructed patients during the telephone follow-up to abstain from smoking and acidic food consumption while taking the medication during the course of the treatment. Patient compliance was observed to be high throughout the process. Our clinic is primarily a H. pylori-specific clinic, where doctors and nurses are dedicated to educating patients about H. pylori treatment and answering all their queries carefully. The patients with underlying medical condition are different as it can have an impact on H. pylori eradication. H. pylori eradication rate in gastritis, gastric ulcers and duodenal ulcers has been reported to be different [32]. Our study only included patients with H. pylori infection who were essentially gastroscopically as well as pathologically confirmed to have chronic gastritis.

The Maastricht VI/Florence consensus and 2022 Chinese national clinical practice guidelines on *H. pylori* eradication treatment clearly state that both bismuth quadruple therapy and high-dose dual therapy could be prescribed for initial and second eradication treatments [1, 33]. In another elegant study, Han et al. compared the efficacy of high-dose diphtherapy and bismuth-containing quadruple therapy as remedial treatment option for *H. pylori*, with no significant difference observed in eradication rates [34]. High-dose diphtherapy can effectively increase the dose and frequency of effective drugs, simplify the treatment regimens, reduce the use of non-essential drugs, and is safe as well as inexpensive. It can also improve patient acceptance and compliance, reduce the rate of *H. pylori* drug resistance, and has emerged as a new and effective therapy against *H. pylori* infection [34]. This observation is also reflected in our study results that high-dose diphtherapy and bismuth-containing quadruple therapy can be potentially used as first-line regimens for *H. pylori* salvage therapy. However, because of the high success rate of initial treatment in this study and relatively small sample size entering remedial therapy, the results of this phase of the study still need to be further validated by additional data from larger studies.

It has been established that identification of strain profiles in different geographic regions and bacterial virulence factors are often associated with risk of severe disease that can provide useful information for personalized eradication therapy for *H. pylori*. Personalized therapy for *H. pylori* is an option and cannot only improve *H. pylori* eradication rates but also significantly reduce antibiotic resistance. We found that the prevalence of *H. pylori* infection in Hainan is relatively high, the number of refractory infections is increasing rapidly, the impact of drug resistance is unclear, and the choice of clinical eradication drug regimens lacks reliable reference. Our team is currently conducting research on the distribution of *H. pylori* culture and drug resistance in Hainan, and we believe that we will be able to obtain results soon. Based on national and international *H. pylori* consensus, local drug sensitivity results and local clinical experience, we have identified our two *H. pylori* eradication protocols (dual therapy with IA at high dose versus IAFB quadruple therapy). Antibiotic susceptibility testing (AST)-guided triple therapy and triple therapy can on average, provide moderate to large clinical benefit in comparison with empiric therapy [1, 33]. However, in China, antibiotic susceptibility can markedly reduce the feasibility and clinical availability, and not all health care facilities possess the capacity to offer these tests. Thus, routine use of the test may lead to inequitable access to the healthcare. Overall, the Guideline Development Group (GDG) concluded that the benefits of using AST to guide the therapy can outweigh the potential adverse effects and that its use should be encouraged at least in people on second-line therapy or with a history of treatment failure [33].

The study has few limitations. Firstly, the study is characterized by single center, small sample size and hence cannot be generalized. Secondly, due to the limitations associated with the culture of *H. pylori*, drug resistance analysis and genotyping analysis, monitoring of CYP2C19, gastric pH and blood concentration of both drugs were not performed, and all the above indicated factors might also affect the final results.

In conclusion, IA dual therapy was found to be equally effective and safer and less costly in comparison to IAFB quadruple therapy. Therefore, these therapies can be considered as first-line regimens for empirical treatment.

## Data Availability

The datasets generated and/or analyzed during the current study can be obtained from the corresponding author upon reasonable request.

## References

[1] Malfertheiner P, Megraud F, Rokkas T, Gisbert JP, Liou JM, Schulz C (2022). European Helicobacter and Microbiota Study group. Management of Helicobacter pylori infection: the Maastricht VI/Florence consensus report. Gut.

[2] Fallone CA, Chiba N, Zanten SV, Fischbach L, Gisbert JP, Hunt RH (2016). The Toronto Consensus for the treatment of Helicobacter pylori infection in adults. Gastroenterology.

[3] The Helicobacter pylori Group of the Chinese Medical Association, Gastroenterology Branch (2022). Sixth national consensus report on the management of Helicobacter pylori infection (non-elimination treatment part). Chin J Gastroenterol.

[4] Liu WZ, Xie Y, Lu H, Cheng H, Zeng ZR, Zhou LY (2018). Fifth Chinese National Consensus Report on the management of Helicobacter pylori infection. Helicobacter.

[5] Yang X, Wang JX, Han SX, Gao CP (2019). High dose dual therapy versus bismuth quadruple therapy for Helicobacter pylori eradication treatment: a systematic review and meta-analysis. Medicine.

[6] Gao CP, Zhang D, Zhang T, Wang JX, Han SX, Graham DY (2020). PPI-amoxicillin dual therapy for Helicobacter pylori infection: an update based on a systematic review and meta-analysis. Helicobacter.

[7] Huang Q, Shi Z, Cheng H, Ye H, Zhang X (2021). Efficacy and safety of modified dual therapy as the first-line regimen for the treatment of Helicobacter pylori infection: a Meta-analysis of Randomized controlled trials. J Clin Gastroenterol.

[8] Seo KA, Lee SJ, Kim KB, Bae SK, Liu KH, Kim DH (2012). Ilaprazole, a new proton pump inhibitor, is primarily metabolized to ilaprazole sulfone by CYP3A4 and 3A5. Xenobiotica.

[9] Wang H, Shao F, Liu X, Xu W, Ou N, Qin X (2019). Efficacy, safety and pharmacokinetics of ilaprazole infusion in healthy subjects and patients with esomeprazole as positive control. Br J Clin Pharmacol.

[10] Periclou AP, Goldwater R, Lee SM, Park DW, Kim DY, Cho KD (2000). A comparative pharmacodynamic study of IY-81149 versus omeprazole in patients with gastroesophageal reflux disease. Clin Pharmacol Ther.

[11] Shen T, Jiang X, Jin Z, Ji Q, Li C, Li Q (2020). The study of intestinal absorption and biodistribution in vivo of proton pump inhibitors. Eur J Pharm Biopharm.

[12] Niu M, Zhou Y, Xie Y, Li X, Tian Y, Yao L (2022). Comparison of the dual therapy of Ilaprazole-Amoxicillin and the Bismuth Quadruple Therapy of Ilaprazole-Amoxicillin-Furazolidone-Bismuth glycyrrhizinate for eradication of Helicobacter pylori. Front Pharmacol.

[13] Jin Y, Zhang S, Pan J, Yue M, Zhang G, Yao D (2019). Comparison of efficacy and safety of ilaprazole and esomeprazole both in initial treatment regimen and retreatment regimen of Helicobacter pylori infection in chronic gastritis. Pharmazie.

[14] Yang JC, Lin CJ, Wang HL, Chen JD, Kao JY, Shun CT (2015). High-dose dual therapy is superior to standard first-line or rescue therapy for Helicobacter pylori infection. Clin Gastroenterol Hepatol.

[15] Song Z, Zhou L, Xue Y, Suo B, Tian X, Niu Z (2020). A comparative study of 14-day dual therapy (esomeprazole and amoxicillin four times daily) and triple plus bismuth therapy for first-line Helicobacter pylori infection eradication: a randomized trial. Helicobacter.

[16] Butt J, Varga MG, Blot WJ, Teras L, Visvanathan K, Le Marchand L (2019). Serologic response to Helicobacter pylori proteins associated with risk of colorectal cancer among diverse populations in the United States. Gastroenterology.

[17] Yang Q, He C, Hu Y, Hong J, Zhu Z, Xie Y (2023). 14-day pantoprazole-and amoxicillin-containing high-dose dual therapy for Helicobacter pylori eradication in elderly patients: a prospective, randomized controlled trial. Front Pharmacol.

[18] Savoldi A, Carrara E, Graham DY, Conti M, Tacconelli E (2018). Prevalence of antibiotic resistance in Helicobacter pylori: a systematic review and meta-analysis in World Health Organization regions. Gastroenterology.

[19] Li C, Shi Y, Suo B, Tian X, Zhou L, Song Z (2021). PPI-amoxicillin dual therapy four times daily is superior to guidelines recommended regimens in the Helicobacter pylori eradication therapy within Asia: a systematic review and meta-analysis. Helicobacter.

[20] Sakurai Y, Mori Y, Okamoto H, Nishimura A, Komura E, Araki T (2015). Acid-inhibitory effects of vonoprazan 20 mg compared with esomeprazole 20 mg or rabeprazole 10 mg in healthy adult male subjects - a randomised open-label cross-over study. Aliment Pharmacol Ther.

[21] Hu Y, Xu X, Ouyang YB, He C, Li NS, Xie C (2022). Altered gut microbiota and short-chain fatty acids after Vonoprazan-Amoxicillin Dual Therapy for Helicobacter pylori Eradication. Front Cell Infect Microbiol.

[22] Mukherjee S, Madathil SA, Ghatak S, Jahau L, Pautu JL, Zohmingthanga J (2020). Association of tobacco smoke–infused water (tuibur) use by Mizo people and risk of Helicobacter pylori infection. Environ Sci Pollut Res Int.

[23] Hu CT, Tung C, Lin CJ, Kuo IJ, Lin BR, Wang HL (2017). Efficacy of high-dose dual therapy versus bismuthcontaining quadruple therapy for first-line treatment of helicobacter pylori infection and an interim report of multi-center, randomized control study. Gastroenterology.

[24] Tai WC, Liang CM, Kuo CM, Huang PY, Wu CK, Yang SC (2019). A 14 day esomeprazole-and amoxicillin-containing high-dose dual therapy regimen achieves a high eradication rate as first-line anti-helicobacter pylori treatment in Taiwan: a prospective randomized trial. J Antimicrob Chemother.

[25] Gao W, Ye H, Deng X, Wang C, Xu Y, Li Y (2020). Rabeprazole-amoxicillin dual therapy as first-line treatment for H pylori eradication in special patients: a retrospective, real-life study. Helicobacter.

[26] Dore MP, Lu H, Graham DY (2016). Role of bismuth in improving Helicobacter pylori eradication with triple therapy. Gut.

[27] Ford AC, Malfertheiner P, Giguere M, Santana J, Khan M, Moayyedi P (2008). Adverse events with bismuth salts for Helicobacterpylori eradication: systematic review and meta-analysis. World J Gastroenterol.

[28] Alkim H, Koksal A, Boga S, Sen I, Alkim C (2017). Role of bismuth in the eradication of Helicobacter pylori. Am J Ther.

[29] Sun Q, Liang X, Zheng Q, Liu W, Xiao S, Gu W (2010). High efficacy of 14-day triple therapy-based, bismuth-containing quadruple therapy for initial Helicobacter pylori eradication. Helicobacter.

[30] Liou JM, Fang YJ, Chen CC, Bair MJ, Chang CY, Lee YC (2016). Taiwan Gastrointestinal Disease and Helicobacter Consortium. Concomitant, bismuth quadruple, and 14-day triple therapy in the first-line treatment of Helicobacter pylori: a multicentre, open-label, randomised trial. Lancet.

[31] Attumi TA, Graham DY (2014). High-dose extended-release lansoprazole (dexlansoprazole) and amoxicillin dual therapy for Helicobacter pylori infections. Helicobacter.

[32] Gao W, Cheng H, Hu FL, Lü NH, Xie Y, Sheng JQ (2012). [Ilaprazole based bismuth-containing quadruple regimen for the first-line treatment of Helicobacter pylori infection: a multicenter, randomized, controlled clinical study]. Zhonghua Yi Xue Za Zhi.

[33] Zhou L, Lu H, Song Z, Lyu B, Chen Y, Wang J, on behalf of Helicobacter Pylori Study Group of Chinese Society of Gastroenterology (2022). 2022 chinese national clinical practice guideline on Helicobacter pylori eradication treatment. Chin Med J (Engl).

[34] Bi H, Chen X, Chen Y, Zhao X, Wang S, Wang J (2022). Efficacy and safety of high-dose esomeprazole–amoxicillin dual therapy for Helicobacter pylori rescue treatment: a multicenter, prospective, randomized, controlled trial. Chin Med J (Engl).

